# Poor Applicability of Currently Available Prognostic Scoring Systems for Prediction of Outcome in *KIT* D816V-Negative Advanced Systemic Mastocytosis

**DOI:** 10.3390/cancers16030593

**Published:** 2024-01-30

**Authors:** Nicole Naumann, Martina Rudelius, Johannes Lübke, Deborah Christen, Jakob Bresser, Karl Sotlar, Georgia Metzgeroth, Alice Fabarius, Wolf-Karsten Hofmann, Jens Panse, Hans-Peter Horny, Nicholas C. P. Cross, Andreas Reiter, Juliana Schwaab

**Affiliations:** 1 Hematology and Oncology, Medical Faculty Mannheim, Heidelberg University, 68167 Mannheim, Germany; nicole.naumann@medma.uni-heidelberg.de (N.N.);; 2 Institute of Pathology, Ludwig-Maximilian-University, 80337 Munich, Germany; 3 Department of Oncology, Haematology, Haemostaseology and Stem Cell Transplantation, University Hospital RWTH Aachen, 52074 Aachen, Germany; 4 Center for Integrated Oncology (CIO), Aachen, Bonn, Cologne, Düsseldorf (ABCD), 52074 Aachen, Germany; 5 Institute of Pathology, University Hospital Salzburg, Paracelsus Medical University, 5020 Salzburg, Austria; 6 Wessex Genomics Laboratory Service, Salisbury SP2 8BJ, UK; 7 Faculty of Medicine, University of Southampton, Southampton SO17 1BJ, UK

**Keywords:** advanced systemic mastocytosis, *KIT* D816V-negative, *KIT* D816H, *KIT* D816Y, *KIT* mutation-negative

## Abstract

**Simple Summary:**

Most patients (>95%) with systemic mastocytosis (SM) carry a mutation in the *KIT* gene (*KIT* D816V). Especially aggressive forms of SM are associated with pronounced clinical symptoms, blood count abnormalities, additional mutations in other genes and a shortened survival. Only a small minority of patients with SM have another mutation on position D816 (e.g., D816H) or do not harbor any mutation in *KIT*, and data on these small subgroups are scarce. The aim of our study was to characterize these rare subgroups: we examined 7 SM patients with either *KIT* D816H or *KIT* D816Y and 12 SM patients without any *KIT* mutation. We found that (a) both groups frequently appear as mast cell leukemia (the most aggressive SM subgroup), (b) those patients cannot be assessed using conventional risk scores, (c) response to treatment is poor and (d) overall survival is worse than in *KIT* D816V-positive SM.

**Abstract:**

Within our nationwide registry, we identified a *KIT* D816V mutation (*KIT* D816V^pos.)^ in 280/299 (94%) patients with advanced systemic mastocytosis (AdvSM). Age, cytopenias and the presence of additional somatic mutations confer inferior overall survival (OS). However, little is known about the characteristics of *KIT* D816V-negative (D816V^neg.^) AdvSM. In 19 D816V^neg.^ patients, a combination of clinical, morphological and genetic features revealed three subgroups: (a) *KIT* D816H- or Y-positive SM (*KIT* D816H/Y^pos.^, *n* = 7), predominantly presenting as mast cell leukemia (MCL; 6/7 patients), (b) MCL with negative *KIT* sequencing (*KIT*^neg.^ MCL, *n* = 7) and (c) *KIT*^neg.^ SM with associated hematologic neoplasm (*KIT*^neg.^ SM-AHN, *n* = 5). Although >70% of patients in the two MCL cohorts (*KIT* D816H/Y^pos.^ and *KIT*^neg.^) were classified as low/intermediate risk according to prognostic scoring systems (PSS), treatment response was poor and median OS was shorter than in a *KIT* D816V^pos.^ MCL control cohort (*n* = 29; 1.7 vs. 0.9 vs. 2.6 years; *p* < 0.04). The *KIT*^neg.^ SM-AHN phenotype was dominated by the heterogeneous AHN (low mast cell burden, presence of additional mutations) with a better median OS of 4.5 years. We conclude that (i) in MCL, negativity for D816V is a relevant prognostic factor and (ii) PSS fail to correctly classify D816V^neg.^ patients.

## 1. Introduction

Systemic mastocytosis (SM) is a rare myeloid neoplasm with an accumulation of neoplastic mast cells (MC) in various tissues, most often in bone marrow (BM), skin and the gastrointestinal tract. The disease is subcategorized into indolent SM (ISM), smoldering SM (SSM), bone marrow mastocytosis (BMM) and advanced SM (AdvSM), the latter comprising SM with an associated hematologic/myeloid neoplasm (SM-AHN/SM-AMN), aggressive SM (ASM) and mast cell leukemia (MCL) [1]. ISM is associated with a normal life expectancy whereas survival in AdvSM is dependent on subtype and ranges between 1.5 and 4 years [1].

Mast cells play an important role in the immune system and are directly involved in allergic reactions, stress and tumor growth and their degranulation may lead to a plethora of different symptoms [2,3]. Their pro- and antitumor potential in general is widely discussed, and a complete depletion of mast cells is regarded as a life-threatening condition. The clonal constitutive activation of mast cells in SM may also lead to a multitude of symptoms affecting different organ systems which sometimes makes the diagnosis challenging.

Cytopenias (anemia < 10 g/dL, platelets < 100 × 10^9^/L, neutrophils < 1 × 10^9^/L), hepatomegaly with impaired liver function (hypoalbuminemia, elevated alkaline phosphatase, portal hypertension, splenomegaly with hypersplenism, ascites), malabsorption with weight loss and large osteolytic lesions constitute the so-called C-findings indicating organ damage. C-findings are diagnostic criteria for ASM but not for SM-AHN and MCL, but they are in fact identified in the majority of patients with SM-AHN and MCL. Additional somatic mutations are present in 60–80% of patients with AdvSM of which mutations in *SRSF2*, *ASXL1*, *RUNX1*, *NRAS* and *DNMT3A* have been associated with poor prognosis [4,5,6]. Several prognostic scoring systems have recently been established variably comprising clinical (age, organomegaly), laboratory (anemia, thrombocytopenia, beta-2-microglobulin and alkaline phosphatase) and genetic (additional somatic mutations) characteristics [4,5,6,7,8].

Depending on the SM subtype, mutations in the receptor tyrosine kinase *KIT*, typically *KIT* D816V, are observed in 80% to over 90% of patients. *KIT* D816V^neg.^ SM/AdvSM comprises alternative mutations at position 816 (e.g., D816H or Y, *KIT* D816H/Y^pos.^) and the absence of any mutation in the coding sequence of *KIT* (*KIT*^neg.^) [9,10,11]. Across all SM subtypes, *KIT*^neg.^ SM is identified at a frequency of 5–10%. However, its prevalence is higher in rare subtypes including MCL (10–20%), well-differentiated systemic mastocytosis (WDSM) (approximately 60–70%) [12,13], myelomastocytic leukemia (> 90%) and mast cell sarcoma (MCS, approx. 90%) [14,15]. Due to limited knowledge, we sought to evaluate the clinical, genetic and prognostic characteristics of patients with *KIT* D816V^neg.^ AdvSM and MCS.

## 2. Patients and Methods

All patients were registered with the “German Registry on Disorders of Eosinophils and Mast cells” (GREM) and gave written informed consent. Detailed information on clinical, morphological and laboratory parameters are presented in Table 1a,b and Appendix A, Table A1. All patients were diagnosed and subtyped as SM according to the 2016 WHO classification. The study design adhered to the tenets of the Declaration of Helsinki and was approved by the responsible institutional review boards.

### 2.1. Cytomorphology and Histomorphology

All biopsies were evaluated by a reference pathologist (M.R., H.-P.H, K.S.) of the ‘European Competence Network on Mastocytosis’ (ECNM). Mast cell morphology was analyzed in BM aspirate smears stained with May–Grünwald–Giemsa (Carl Roth GmbH, Karlsruhe, Germany) and toluidine blue (Merck/Sigma-Aldrich, Darmstadt, Germany). BM trephine biopsy specimens were decalcified for 8 h, fixed in 4% formalin (Carl Roth GmbH, Karlsruhe, Germany) and paraffin-embedded. Sections were stained with hematoxylin (Carl Roth GmbH, Karlsruhe, Germany) and eosin (Carl Roth GmbH, Karlsruhe, Germany), Giemsa (Carl Roth GmbH, Karlsruhe, Germany), Gömöri´s silver impregnation (Merck/Sigma-Aldrich, Darmstadt, Germany) and naphthol AS-D chloracetate esterase (Merck/Sigma-Aldrich, Darmstadt, Germany).

### 2.2. Immunohistochemistry

Immunohistochemistry was performed on the fully automated Ventana Benchmark platform (Roche Diagnostics, Mannheim, Germany) using the Ultraview DAB IHC detection kit (Roche Diagnostics, Mannheim, Germany) for visualization. The following antibodies were used: CD117 (Agilent, Santa Clara, CA, USA), CD25 (Leica, Deer Park, TX, USA), CD2 (Leica), MZT (Zytomed, Berlin, Germany), CD30 (Thermo Fisher Scientific, Waltham, MA, USA), CD20 (Agilent, Santa Clara, CA, USA), CD138 (Zytomed, Berlin, Germany), Kappa (Agilent, Santa Clara, CA, USA), Lambda (Agilent, Santa Clara, CA, USA) and CD5 (Leica Biosystems, Nussloch, Germany).

### 2.3. Microdissection

To exclude false negative *KIT* mutation analysis in patients with a low mast cell burden (SM-AHN, patients #15–#19), we performed microdissection of mast cell aggregates from paraffin-embedded BM biopsy samples for 4/5 (#15, #16, #17, #19) patients.

Prior to manual dissection from the slides, the tissue was deparaffinized and stained with H & E (Carl Roth GmbH, Karlsruhe, Germany). For manual microdissection, compact mast cell infiltrates were identified by a pathologist and the region of interest of the FFPE tissue was scraped into Eppendorf tubes for subsequent DNA/RNA isolation. This method enriched tumor cells, and the tumor cell content was at least 70%. Following the manufacturer’s protocol, we prepared a DNA library using a hybrid capture-based TruSight Oncology 500 DNA/RNA NextSeq Kit (Illumina, San Diego, CA, USA). Using the unique molecular identifiers (UMIs) in the TruSight Oncology 500 (TSO 500) (Illumina, San Diego, CA, USA), we determined the unique coverage of each position and reduced background noise. We analyzed sequencing data for genomic alterations, including SNVs, CNVs and fusions. SNVs and small indels with a variant allele frequency (VAF) of less than 2% were excluded.

### 2.4. Mutation Analysis

Quantitative assessment of the expressed allele burden (EAB) at the RNA level was performed by allele-specific reverse-transcriptase quantitative polymerase chain reaction (RT-qPCR), as previously described [9]. Qualitative assessment of *KIT* D816H/Y was performed by Sanger sequencing of *KIT* exon 17 from peripheral blood (PB) or BM according to standard procedures.

For analysis of alternative *KIT* and additional somatic mutations, Next-Generation Deep Amplicon Sequencing (NGS) by 454 FLX amplicon chemistry (Roche, Penzberg, Germany) or library preparation based on the TruSeq Custom Amplicon Low Input protocol (Illumina, San Diego, CA) followed by sequencing on the MiSeq instrument (Illumina, San Diego, CA, USA) was performed in 18/19 patients. The sequencing panel includes a standard myeloid gene panel covering 18 recurrently mutated genes in myeloid neoplasms. Gene mutations were annotated using the appropriate reference sequence (Ensembl release 85: July 2016).

From 12/12 patients without a mutation in *KIT*, gDNA isolated from PB and/or BM (including patient #18) was used for NGS analysis of all *KIT* exons. The mean coverage was 1500 reads (400–8819). In two patients, whole genome and whole transcriptome sequencing was applied. DNA sequencing was performed using library preparation by ‘TruSeq DNA PCR-Free’ and sequencing by NovaSeq6000 (Illumina, San Diego, CA, USA). RNA sequencing was performed by RNA-Seq (Illumina TruSeq Stranded Total RNA KIT; Unique Dual Indices; Illumina, San Diego, CA, USA). DNA reads were aligned against the human reference genome (hg19); RNA reads were used to detect potential fusion transcripts (Arriba, Manta, STAR-Fusion).

### 2.5. Comparison to Control Group

For classification, risk stratification and evaluation of prognostic factors among *KIT*^neg.^ and *KIT* D816H/Y^pos.^ patients, we utilized a control group of *KIT* D816V^pos.^ AdvSM patients (*n* = 118; SM-AHN, *n* = 89; MCL-(AHN), *n* = 29) with known clinical, laboratory and genetic characteristics (Table 1a,b and Table 2).

### 2.6. Statistical Analyses

All statistical analyses were performed using IBM^®^ SPSS statistics (version 25.0, IBM-Corporation, Armonk, NY, USA) or GraphPad Prism (version 8, GraphPad, San Diego, CA, USA). Survival probabilities were calculated by the Kaplan–Meier method and were determined from the date of diagnosis to death or the date of last contact (if alive). Overall survival (OS) was defined as the time from AdvSM diagnosis to death from any cause. In patients undergoing allo-SCT, progression-free survival (PFS) was defined as the time from allogeneic stem cell transplantation (SCT) to disease progression or death from any cause. A *p* < 0.05 was considered as statistically significant.

## 3. Results

**Figure 1:**
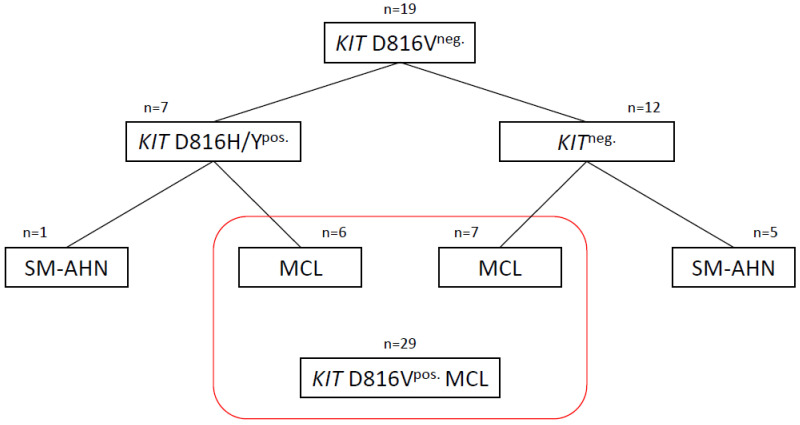
Overview of patient cohorts. Clear dominance of MCL phenotype in *KIT* D816H/Y^pos.^ and *KIT*^neg.^ patients. MCL patients were subsequently compared to a *KIT* D816V^pos.^ MCL control cohort from the registry (see red box). Abbreviations: MCL, mast cell leukemia; AHN, associated hematologic neoplasm; SM, systemic mastocytosis; neg., negative; pos., positive.

From almost 300 advSM patients collected in the GREM, we identified 19 patients without a *KIT* D816V mutation. Seven patients carried an alternative mutation on locus *KIT* D816 (D816H, *n* = 4; D816Y, *n* = 3), whereas no mutation in the *KIT* gene was detected in 12 patients (*KIT*^neg.^). While almost all (6/7, 86%) patients of the *KIT* D816H/Y^pos.^ cohort were diagnosed with MCL, the *KIT*^neg.^ subgroup included MCL (7/12, 58%) and SM-AHN (5/12, 42%) patients (Figure 1).

### 3.1. KIT D816H/Y^pos.^ Patients

*Clinical features.* The median age of *KIT* D816H/Y^pos.^ patients (*n* = 7) was 58 years (range 37–69, m/f: 3/4). The median BM MC infiltration was 50% (range 20–75%); other notable BM features included eosinophilia (6/7, 86%) and fibrosis (4/7, 57%; grade 2, 2/7, grade 3, 2/7). Blood counts revealed mild leukocytosis > 10 and <16 × 10^9^/L (2/7, 29%) and anemia < 10 g/dL (4/7, 57%, one patient transfusion dependent), while thrombocytopenia < 100 G/L was absent in all seven patients. Eosinophilia > 0.4 × 10^9^/L was frequent (5/7, 82%; median 1 × 10^9^/L, range 0–39) while monocytosis > 1 × 10^9^/L was rare (1/7; 14%, median 0.23 × 10^9^/L, range 0.03–1.3). Other C-findings included ascites (5/7, 71%), elevated alkaline phosphatase (4/7, 57%) and hypoalbuminemia (1/7, 14%). Involvement of the gastrointestinal tract and skin (0/7) was rare (1/7, 14%). The median serum tryptase level was 193 µg/L (range 90–1300). Consequently, morphologic subtypes included MCL ± AHN (6/7, 86%; +MCS, 2/7, 29%; with WDSM phenotype, 1/7, 14%) and SM-AHN (1/7, 14%). Clinically relevant anaphylaxis (1/7, 14%) was rare and no other relevant MC-triggered symptoms were noted. 

#### 3.1.1. Molecular Findings

**Figure A1:**
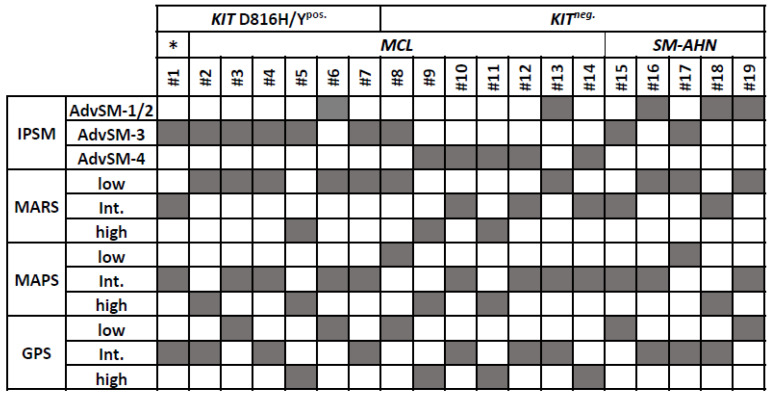
Assignment of risk scores for individual patients. The majority of patients were grouped as low- or intermediate-risk within the molecular risk scores for advanced systemic mastocytosis (MARS, MAPS, GPS). IPSM was the most effective score in discriminating between low risk (AdvSM-1/2) and higher risk profile (AdvSM-3/4). Abbreviations: IPSM, International Prognostic Scoring System for Mastocytosis; GPS, Global Prognostic Score for Mastocytosis; MAPS, Mayo Alliance Prognostic System; MARS, Mutation-Adjusted Risk Score for Advanced Mastocytosis, MCL, mast cell leukemia; SM, systemic mastocytosis; int., intermediate; *, SM-AHN.

Additional somatic high-risk mutations (HRM) in *SRSF2*, *ASXL1*, *RUNX1* (*S*/*A*/*R* gene panel) [6], *NRAS* or *DNMT3A* [4,16] were identified in 2/7 (29%) patients (*SRSF2*, *n* = 1; *NRAS*, *n* = 1) and other somatic mutations (*TET2*, *JAK2*, and *CBL*) [5] were observed in two patients (29%; *CBL/TET2*, 1/7; *JAK2* V617F, 1/7). Due to the paucity of leukocytosis ≥ 16 × 10^9^/L (0/7), skin involvement (0/7) and HRM (2/7), almost all patients were low/intermediate risk according to current prognostic scoring systems for AdvSM (IPSM, 7/7; MARS, 6/7; MAPS, 5/7; GPS, 6/7) (Figure A1).

#### 3.1.2. Treatment

**Figure 2:**
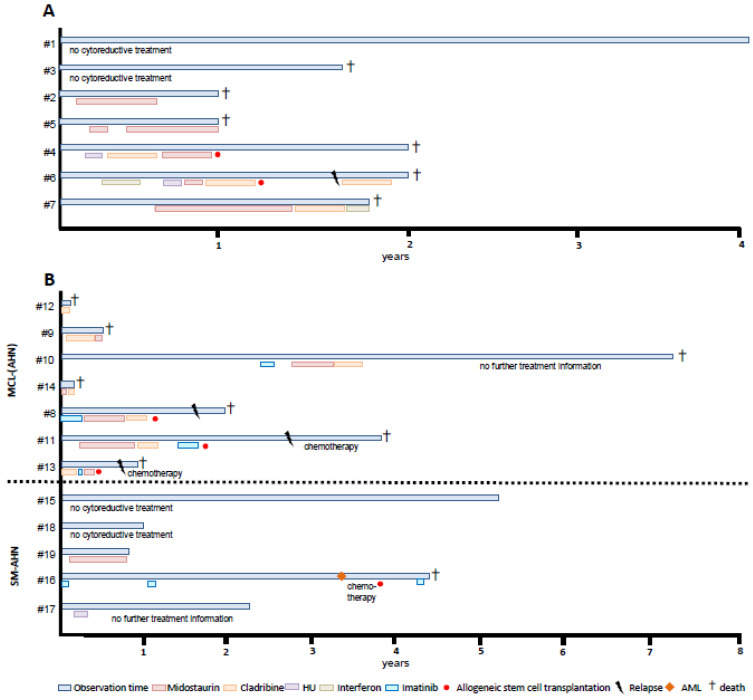
Individual course of *KIT* D816H/Y^pos.^ patients (**A**) and *KIT*^neg.^ patients (**B**) Detailed information on treatment sequences for *KIT* D816H/Y^pos.^ and *KIT*^neg.^ patients. *KIT*^neg.^ patients were further subdivided into MCL and SM-AHN phenotypes. Treatment sequences are depicted in different colors. Six patients underwent allogeneic stem cell transplantation with rapid relapse. Inappropriate treatment response led to death in 14/19 patients. Abbreviations: MCL, mast cell leukemia; AHN, associated hematologic neoplasm; SM, systemic mastocytosis †, death.

Treatment comprised midostaurin (5/7, 71%) with or without sequential cladribine (3/7, 43%) and supportive care only (2/7, 29%; G-CSF, local radiation). The response to treatment was only partially effective and of short duration. Allogeneic SCT was performed in 2/7 (29%) patients who relapsed early and deceased at months +10 and +12 after transplant (Figure 2). Despite a predominantly low/intermediate risk profile, the median OS was only 22 months (range 12–49).

### 3.2. KIT^neg.^ Patients

Due to significant clinical and morphological differences, two subgroups were considered (Table 3): MCL ± AHN (7/12, 58%) and SM-AHN (5/12, 42%).

#### 3.2.1. Cytomorphology and Histomorphology

**Figure 3:**
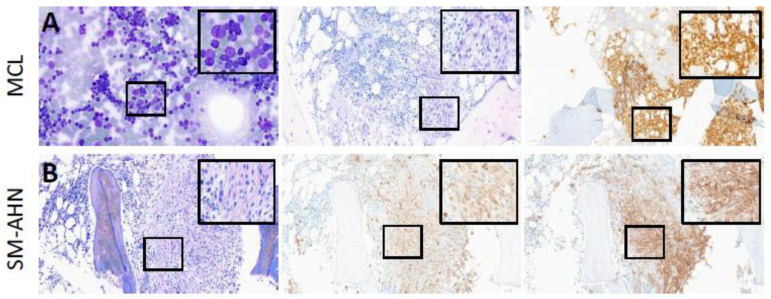
Histomorphology (2.5× and 40× magnification) of a patient with MCL (**A**) and SM-AHN (**B**). (**A**) Bone marrow aspirate shows an increase of atypical mast cells (≥20%); in bone marrow core biopsies dense infiltrates of hypogranular mast cells with positivity of CD25 were present. (**B**) Bone marrow core biopsies showed diagnostic compact mast cell infiltrates of spindle-shaped mast cells with expression of mast cell tryptase and aberrant expression of CD25. Abbreviations: MCL, mast cell leukemia; AHN, associated hematologic neoplasm; SM, systemic mastocytosis.

The BM aspirates of patients with *KIT*^neg.^ MCL exhibited atypical MC with round nuclei rather than a spindle-shaped morphology (Figure 3A). Two cases presented a well-differentiated phenotype with prominent cytoplasmic granulation, and only a few neoplastic MCs displayed aberrant expression of CD25. In all BM biopsies, multifocal dense infiltrates with aggregates of ≥15 MC were detected, often localized paratrabecularly. Reticulin fibers were increased, consistent with a myelofibrosis grade 1 (MF1) in the majority of cases. Of the five cases diagnosed with *KIT*^neg.^ SM-AHN, two patients were classified as ISM with associated MDS and multilineage dysplasia (MDS-MLD), one patient was diagnosed with SM- acute myeloid leukemia (AML) and one patient fulfilled criteria for each associated myelofibrosis (SM-MF) and MDS/MPN, respectively. Striking features included: (a) WDSM with a solitary dense infiltrate, diffuse interstitial MC hyperplasia and normal immunophenotype in one patient (case 15) and (b) atypical, spindle-shaped MC exhibiting aberrant CD25 expression without formation of dense clusters in one patient (case 18) (Figure 3B).

#### 3.2.2. Molecular Findings

Additional somatic high-risk mutations (HRM) in the *S*/*A*/*R* gene panel [6], *NRAS* or *DNMT3A* [4,16] were identified in 4/12 (33%) patients (*ASXL1*, *n* = 2; *SRSF2*, *n* = 1; *DNMT3A*, *n* = 1; *RUNX1*, *n* = 1). Other somatic mutations (*TET2*, *JAK2*, *KRAS*, *CSF3R*, *U2AF1*, *CBL*, *FLT3*-ITD) [5] were observed in 6/12 (50%) patients.

#### 3.2.3. Treatment

Treatment comprised TKI (MCL, 6/7 and SM-AHN, 2/5; midostaurin 6/7 and 1/5; imatinib, 3/7 and 1/5) with or without sequential cladribine (6/7 and 0/5) and intensive chemotherapy (0 and 1/5) without significant or durable response. Following allogeneic SCT (3/7 and 1/5), all patients died (months +5, +6, +7, +24) due to progressive disease (Figure 2). According to risk scoring systems of AdvSM, the majority of patients were low or intermediate (IPSM, 2/7 and 5/5; MARS, 5/7 and 5/5; MAPS, 5/7 and 4/5; GPS, 4/7 and 5/5) (Figure A1). The median OS was significantly different (0.9 versus 4.5 years, *p* = 0.037).

### 3.3. Commonalities and Differences between the Two Cohorts of KIT^neg.^ AdvSM

**Figure 4:**
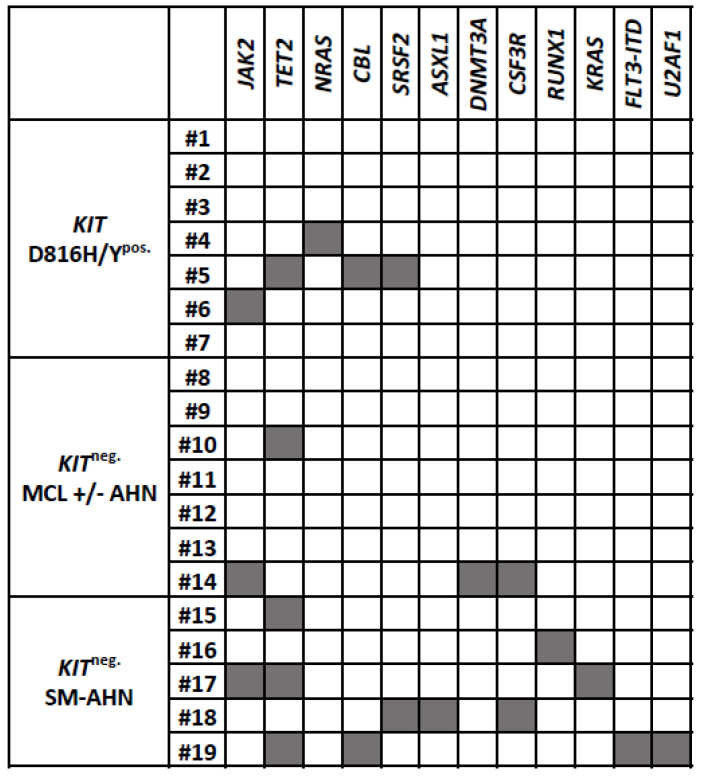
Mutation profile of individual patients. Most additional somatic mutations were detected in *KIT* D816V-negative SM-AHN patients. Mutations detected in genes were involved in epigenetics (*TET2*, *ASXL1* and *DNMT3A*), cell signaling (*KRAS*, *NRAS*, *CBL*, *JAK2* and *CSF3R*), transcription regulation (*RUNX1*) and mRNA splicing (*SRSF2* and *U2AF1*). Compared to other chronic myeloid neoplasms, mutations in high-risk genes (e.g., *SRSF2*, *RUNX1*, *ASXL1*) were less common. Abbreviations: SM, systemic mastocytosis; MCL, mast cell leukemia; AHN, associated hematologic neoplasm.

The median age (56/62 years, range 27–69) and the m/f ratio were not different (5/7 and 3/5). Significant differences were identified for BM MC infiltration (median 80% versus 20%, *p* = 0.0013), serum tryptase levels (median 451 µg/L versus 34 µg/L, *p* = 0.0025), hemoglobin < 10 g/dL (57% versus 0%; median 9.3 g/dL versus 13 g/dL, *p* = 0.01) and additional somatic mutations (43% and 100%: HRM 2/7 and 2/5; other somatic mutations 2/7 and 4/5). See Figure 4 and Appendix A, Table A2 for detailed molecular features. Although present in some patients, no significant differences between the two subtypes were found for leukocytosis, monocytosis, eosinophilia, alkaline phosphatase (AP), albumin and ascites.

### 3.4. Analysis of Three MCL Cohorts

**Figure 5:**
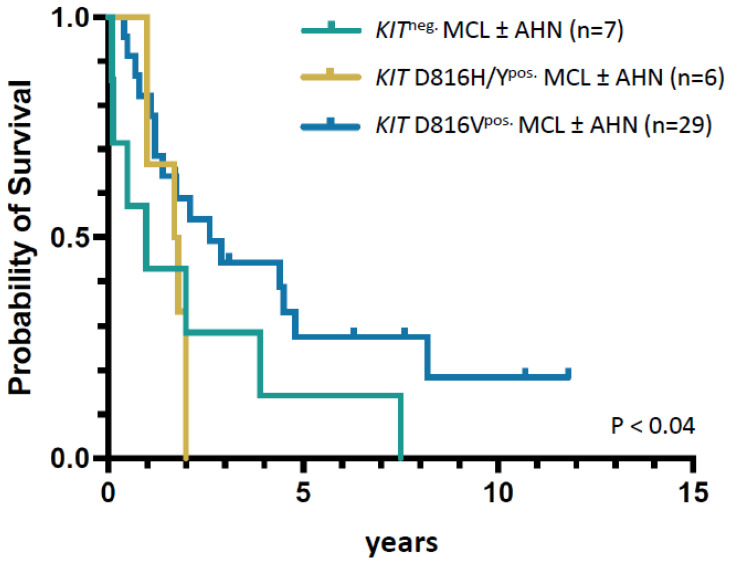
Kaplan–Meier overall survival of patients with mast cell leukemia and presence of *KIT* D816V, *KIT* D816H/Y or absence of a *KIT* mutation. *KIT* D816H/Y-positive MCL patients are associated with the worst outcome. *KIT* D816V-positive MCL patients have the worst prognosis within all D816V-positive SM subgroups, but still have a better overall survival than *KIT* D816V-negative MCL patients. Abbreviations: MCL, mast cell leukemia; AHN, associated hematologic neoplasm.

Based on registry data, the two MCL cohorts (*KIT* D816H/Y^pos.^, *n* = 6; *KIT*^neg.^ MCL ± AHN, *n* = 7) were compared with a *KIT* D816V^pos.^ MCL cohort (*n* = 29). The most notable significant differences (*KIT* D816V^pos.^ versus *KIT* D816H/Y^pos.^ versus *KIT*^neg.^ MCL ± AHN) included age (median 68 versus 55 versus 56 years, *p* < 0.02), vitamin B12 levels (median 1597 versus 300 versus 3091 µg/L, *p* < 0.04), HRM (66% versus 33% versus 29%, *p* = 0.03) and survival (2.6 versus 1.7 versus 0.9 years; *p* = 0.04). One patient (#3) had chronic MCL. There were no significant differences regarding BM MC infiltration, serum tryptase levels and blood counts (Table 2 and Figure 5).

## 4. Discussion

The pathogenetic driver mutation *KIT* D816V is detected in >90% of SM patients and is therefore of utmost relevance for the diagnosis of this heterogeneous disease. Its presence and variant allele frequency (VAF) in BM and PB are of paramount importance in assessing the phenotype and overall burden of involvement of MC, but often also non-MC lineages in SM-AHN. Currently available PCR assays are capable of detecting *KIT* D816V VAF with a sensitivity of up to 0.003%, allowing quantification in patients with very low disease burden at diagnosis and also monitoring response and residual *KIT* D816V mutational burden during or after treatment with *KIT* inhibitors, chemotherapy or allogeneic SCT [9,17].

In contrast, very little is known about the clinical phenotype of adult *KIT* D816V^neg.^ SM. It has been primarily associated with MCL, WDSM and MCS [18]. Alternative mutations in *KIT* are recurrently identified at position 816, e.g., D816H, D816N or D816Y [12,19]. In isolated cases, other acquired imatinib-resistant mutations have been identified in coding regions of TK domains 1 and 2 (exons 13 to 18), e.g., I817V in WDSM, D820G in ASM and N822K in SM-AHN [18]. Potentially imatinib-sensitive mutations between exons 8 and 10 may correspond to germline mutations, e.g., S451C, K509I or F522C, which often show a familial aggregation pattern [20,21,22,23,24,25,26,27].

Of 298 patients with AdvSM included in the GREM, we identified 19 *KIT* D816V^neg.^ patients, corresponding to a 94% prevalence of *KIT* D816V in AdvSM. Approximately 35% of *KIT* D816V^neg.^ patients were positive for alternative *KIT* D816 mutations. In seven patients, we identified only D816H and D816Y, which are known recurrent mutations, whereas D816G, D816I, D816T and D816N were identified only in single cases [18]. Similar to what has been described in the literature [18], all but one of the seven patients in our series had an MCL phenotype. Of note, despite the low prevalence of poor prognostic markers such as cytopenias, other C-findings and additional somatic mutations that placed the vast majority of patients in low/medium risk categories in most available prognostic scoring systems, prognosis was poor with a median survival of only 1.8 years. Application of the IPSM risk score delivered the best results: here, the majority of patients were classified into the AdvSM-3 risk group. According to Sperr et al. [8], patients within the AdvSM-3 and AdvSM-4 exhibit a significantly worse OS compared to AdvSM-1 and AdvSM-2 patients. However, none of the patients were classified into the AdvSM-4 subgroup. These findings suggest a potentially better discrimination of non-molecular scoring systems such as the IPSM but also show that patients with a lack of cytopenia(s) are rarely stratified into the high-risk subgroups of molecular PSS and or AdvSM-4, which overall show the worst OS.

In the remaining 12 patients without a mutation at position 816, no alternative mutations were detected in the complete coding sequences of *KIT*. From a clinical perspective, two distinct subgroups emerged. Patients with MCL had a very high MC burden and a poor prognosis despite the absence of additional somatic mutations and a predominantly good/intermediate prognostic risk score using molecular annotated risk scores. Again, IPSM was the best discriminator of OS in this subgroup (Appendix A, Figure A1). All MCL patients were aleukemic with three of seven patients presenting with an immature MC morphology, which was predictive of an inferior OS in multivariate analysis in a retrospective study by Pardanani and colleagues [28].

In contrast, patients in the second cohort were diagnosed with SM and an associated hematological neoplasm (SM-AHN) according to the WHO classification or an associated myeloid neoplasm (SM-AMN) according to the ICC. Interestingly, no cases of chronic myelomonocytic leukemia were diagnosed, despite it being the most common SM-associated AHN based on previous studies [29,30]. Patients from our cohort mainly presented with MDS, MDS/MPN overlap or MPN as associated AHN. In two cases, the diagnosis could only be made through a thorough integration of clinical data and elevated tryptase levels, as either a major histomorphological criterion was lacking or only one minor criterion was present. This highlights the necessity for meticulous morphological analysis supplemented by additional immunohistochemistry and interdisciplinary collaboration when faced with *KIT*^neg.^ SM cases. All patients had a dominating AHN with only a low MC burden and all patients exhibited additional somatic mutations. Nevertheless, the prognosis was significantly better than in patients with the MCL phenotype.

The diagnosis of all subtypes of SM can be notably challenging when the fraction of diagnostic neoplastic cells in the BM is low. Consequently, the application of sensitive diagnostic methods becomes imperative, often necessitating a comprehensive approach involving multiple techniques, such as thorough clinical evaluation, histomorphology, immunohistochemistry and molecular genetics. The updated WHO classification and ICC require the fulfillment of one major criterion and two minor criteria, or alternatively, three minor criteria for the diagnosis of SM [14,15]. One of the minor criteria involves proving the clonality of the MC through the detection of a *KIT* mutation, making the diagnosis challenging in cases lacking this mutation. In an unknown proportion of patients, the presence of two clonally independent diseases seems possible, similar to the recently reported cohort of patients with recurrent concurrent presence of *KIT* D816V^pos.^ SM and *JAK2* V617F^pos.^ MPN in the same individual [31].

In a large series of 92 MCL patients of the ECNM, 73% were *KIT* D816V^pos.^, 11% exhibited alternative *KIT* mutations and 17% were *KIT*^neg.^ [32]. In other series, positivity for *KIT* D816V ranged from 23% to 68% [19,33,34]. We compared the two *KIT* D816H/Y^pos.^/*KIT*^neg.^ MCL groups with a larger cohort of 29 registry patients with *KIT* D816V^pos.^ MCL. Although patients with *KIT* D816V^pos.^ MCL were median >10 years older and had a significantly higher incidence of HRM, median OS was significantly better. This suggests that yet unknown mechanisms beyond MC burden, phenotype and genetic profile contribute to the poor prognosis of the different MCL subtypes. The 13 *KIT*^neg.^/*KIT* D816H/Y^pos.^ MCL patients did not show a significant and durable response to midostaurin or imatinib, and all five allogeneic transplanted patients died within two years after transplantation. *KIT* D816V^neg.^ MCL is therefore a high-risk condition regardless of established prognostic scoring systems, especially molecular annotated risk scores. There are no data yet on the efficacy of newly developed tyrosine kinase inhibitors (TKIs) such as avapritinib or bezuclastinib in *KIT* D816V^neg.^ patients.

Important limitations of our analyses include that elaborate statistical analyses and validations in independent control cohorts could not be performed due to the rarity but also heterogeneity of *KIT* D816V^neg.^ AdvSM. Notwithstanding, most of the few available case reports or small series in the literature lack a comparable thorough clinical, morphological and molecular work-up. The data clearly show that *KIT* D816V^neg.^ AdvSM is similarly heterogeneous to *KIT* D816V^pos.^ AdvSM and that a distinct differentiation between the various subtypes, e.g., MCL and SM-AHN, is of utmost relevance for prognostication and treatment decisions.

## 5. Conclusions

In conclusion, the diagnosis of *KIT*^neg.^ AdvSM demands a high level of expertise and close collaboration between hematologists and hematopathologists to avoid diagnostic pitfalls and ensure optimal patient therapy. The integration of multiple diagnostic modalities and molecular studies is essential to achieve accurate SM diagnosis and provide patients with the most effective treatment options. *KIT*^neg.^ AdvSM can be subdivided into (i) patients with other mutations at codon 816 such as *KIT* D816H/N/Y, most frequently associated with an MCL phenotype, (ii) *KIT*^neg.^ MCL with a very poor prognosis despite the absence of C-findings and HRM (iii) *KIT*^neg.^ SM-AHN with low MC burden and dominating AHN, making SM-specific treatment dispensable and prognosis mainly triggered by AHN. Our data highlight the poor applicability of the majority of current prognostic scoring systems for *KIT*^neg.^ MCL.

## Figures and Tables

**Table 1:** (**a**) Diagnosis of *KIT*^neg.^ and *KIT* D816H/Y^pos.^ patients in comparison with a registry-based control group of *KIT* D816V^pos.^ AdvSM patients. (**b**) Clinical, morphological and laboratory parameters of *KIT* D816H/Y^pos.^ and *KIT*^neg.^ patients in comparison with a registry-based control group of *KIT* D816V^pos.^ AdvSM patients.

**(a)**						
**Variables**	***KIT* D816H/Y^pos.^**	***KIT*^neg.^ AdvSM**	***p*-Value**	***KIT* D816V^pos.^ AdvSM**	***p*-Value** **D816V^pos.^ vs. D816H/Y^pos.^**	***p*-Value** **D816V^pos.^ vs. *KIT*^neg.^**
Number of patients, *n*	7	12	-	118	-	-
Age in years, median (range)	58 (37–69)	60 (27–82)	n.s.	68 (33–87)	0.0047	0.0128
Male, *n* (%)	3 (43)	8 (67)	n.s.	83 (70)	n.s.	n.s.
Diagnosis						
SM-AHN, *n* (%)	1 (14)	5 (41)	n.s.	89 (75)	-	-
MCL ± AHN, *n* (%)	6 (86)	7 (58)	n.s.	29 (25)	-	-
AHN-subtypes, *n* (%)	4 (57)	8 (67)	n.s.	111 (94)		
B-NHL, *n* (%)	1 (25)	-	-			
MDS/MPN, *n* (%)	-	2 (25)	-	28	-	-
CLL, *n* (%)	-	1 (13)	-		-	-
MM, *n* (%)	-	1 (13)	-	1 (1)	-	-
CMML, *n* (%)	1 (25)	-	-	37	-	-
MDS, *n* (%)	-	2 (13)	-	15	-	-
AML, *n* (%)	-	1 (25)	-	5	-	-
CEL, *n* (%)	2 (50)	-	-	10	-	-
MF, *n* (%)	-	1 (13)	-	3 (3)	-	-
CNL, *n* (%)	-	-	-	1 (1)	-	-
ET, *n* (%)	-	-	-	2 (2)	-	-
PV, *n* (%)	-	-	-	1 (1)	-	-
MPN, *n* (%)	-	-	-	7 (6)	-	-
Lymphoma *n* (%)	-	-	-	1 (1)	-	-
**(b)**						
**Variables**	***KIT* D816H/Y^pos.^**	***KIT*^neg.^ AdvSM**	***p*-Value**	***KIT* D816V^pos.^ AdvSM**	***p*-Value** **D816V^pos.^ vs. D816H/Y^pos.^**	***p*-Value** **D816V^pos.^ vs. *KIT*^neg.^**
Number of patients, *n*	7	12	-	118	-	-
C-findings						
Platelets, ×10^9^/L; median (range)	149 (100–242)	95 (24–247)	n.s.	94 (5–800)	n.s.	n.s.
<100 × 10^9^/L, *n* (%)	0 (0)	6 (50)	0.0228	64 (54)	0.005	n.s.
Alkaline phosphatase, U/L; median (range)	117 (48–246)	101 (45–437)	n.s.	198 (53–1730)	n.s.	n.s.
>150 U/L, *n* (%)	2 (28)	4 (33)	n.s.	73 (62)	n.s.	n.s.
Albumin, g/L; median (range)	36 (32–45)	37 (20–43)	n.s.	36 (19–48)	n.s.	n.s.
<34 g/L, *n* (%)	1 (14)	3 (25)	n.s.	38 (32)	n.s.	n.s.
Ascites, *n* (%)	5 (71)	1 (8)	0.0023	53 (45)	n.s.	0.0141
B-findings						
MC-infiltration in BM histology, %; median (range)	50 (20–75)	45 (10–90)	n.s.	30 (5–100)	n.s.	n.s.
Serum tryptase, µg/L; median (range)	193 (90–1300)	147 (18–1150)	n.s.	179 (5–1675)	n.s.	n.s.
Splenomegaly, *n* (%)	6 (86)	7 (58)	n.s.	99 (84)	n.s.	n.s.
Hepatomegaly, *n* (%)	2 (28)	4 (33)	n.s.	57 (48)	n.s.	n.s.
Lymphadenopathy, *n* (%)	3 (43)	5 (41)	n.s.	62 (53)	n.s.	n.s.
Additional SM and/or AHN relevant findings						
Leukocytes, ×10^9^/L; median (range)	8.9 (1.6–13.5)	5 (2–91)	n.s.	11 (1–130)	n.s.	n.s.
Monocytes, %; median (range)	5 (1–15)	6 (0–26)	n.s.	8 (0–42)	n.s.	n.s.
Eosinophils, %, median (range)	11 (0–33)	1 (0–28)	n.s.	4 (0–81)	n.s.	n.s.
Vitamin B12, ng/L; median (range)	322 (129–1526)	406 (303–6000)	n.s.	1575 (144–6000)	0.0154	n.s.
>180 ng/L, *n* (%)	6 (86)	8 (100)	n.s.	94 (100)	n.s.	n.s.
*S*/*A*/*R*/*D*/*N* mutation(s), *n* (%)	2 (29)	4 (33)	n.s.	80 (68)	0.0037	0.0042
Other additional mutations, *n* (%)	4 (57)	6 (50)	n.s.	87 (74)	n.s.	n.s.
Anaphylaxis, *n* (%)	1 (14)	1 (8)	n.s.	n.k.	-	-
Treatment						
Midostaurin, *n* (%)	5 (71)	7 (58)	n.s.	72 (61)	n.s.	n.s.
Cladribine, *n* (%)	3 (43)	6 (50)	n.s.	47 (40)	n.s.	n.s.
Imatinib, *n* (%)	0 (0)	4 (33)	n.s.	2 (2)	n.s.	<0.0001
Outcome						
Follow-up, years, median (range)	1.8 (1–2)	1.5 (0.1–7.5)	n.s.	1.8 (0.04–11.82)	n.s.	n.s.
Death, *n* (%)	6 (86)	8 (67)	n.s.	85 (72)	n.s.	n.s.
Overall survival, median, years	1.8	3.9	n.s.	2.4	n.s.	n.s.

AdvSM, advanced systemic mastocytosis; n.s., not significant; SM-AHN, systemic mastocytosis with an associated hematological neoplasm; MCL, mast cell leukemia; B-NHL, B-cell non-Hodgkin lymphoma; MDS, myelodysplastic syndrome; MPN, myeloproliferative neoplasm; CLL, chronic lymphocytic leukemia; MM, multiple myeloma; CMML, chronic myelomonocytic leukemia; AML, acute myeloid leukemia; CEL, chronic eosinophilic leukemia; MF, myelofibrosis; CNL, chronic neutrophilic leukemia; ET, essential thrombocythemia; PV, polycythemia vera; MC, mast cell; *S*/*A*/*R*/*D*/*N*, *SRSF2*, *ASXL1*, *RUNX1*, *DNMT3A*, *NRAS*.

**Table 2:** Clinical, morphological and laboratory parameters of *KIT* D816H/Y^pos.^, *KIT*^neg.^ and *KIT* D816V^pos.^ MCL patients from the registry (control group).

Variables	*KIT* D816H/Y*^pos.^ MCL*	*KIT* ^neg.^ *MCL*	*p*-Value	*KIT* D816V*^pos.^* *MCL*	*p*-Value D816V^pos.^ vs. D816H/Y^pos.^	*p*-Value D816V^pos.^ vs. *KIT*^neg.^
Number of patients, *n*	6	7	-	29	-	-
Age in years, median (range)	55 (37–69)	56 (27–78)	n.s.	68 (45–86)	0.0104	0.0258
Male, *n* (%)	2 (33)	5 (71)	n.s.	20 (69)	n.s.	n.s.
AHN-subtypes, *n* (%)	3 (50)	3 (43)	n.s.	22 (76)	n.s.	n.s.
MDS/MPN, *n* (%)	0 (0)	1 (33)	-	9 (41)	-	-
CLL, *n* (%)	0 (0)	1 (33)	-	0 (0)	-	-
MM, *n* (%)	0 (0)	1 (33)	-	0 (0)	-	-
CMML, *n* (%)	1 (33)	0 (0)	-	6 (27)	-	-
MDS, *n* (%)	0 (0)	0 (0)	-	5 (23)	-	-
AML, *n* (%)	0 (0)	0 (0)	-	1 (5)	-	-
CEL, *n* (%)	2 (66)	0 (0)	-	1 (5)	-	-
C-findings						
Platelets, ×10^9^/L; median (range)	145 (100–242)	59 (24–238)	n.s.	93 (18–795)	n.s.	n.s.
<100 × 10^9^/L, *n* (%)	0 (0)	5 (71)	0.0045	19 (66)	0.0025	n.s.
Alkaline phosphatase, U/L; median (range)	120 (83–246)	131 (59–437)	n.s.	214 (62–548)	n.s.	n.s.
>150 U/L, *n* (%)	2 (33)	3 (43)	n.s.	21 (72)	n.s.	n.s.
Albumin, g/L; median (range)	36 (32–45)	35 (20–41)	n.s.	34.4 (23.4–46)	n.s.	n.s.
<34 g/L, *n* (%)	1 (17)	3 (43)	n.s.	11 (39)	n.s.	n.s.
Ascites, *n* (%)	5 (83)	1 (14)	0.009	16 (57)	n.s.	n.s.
B-findings						
MC-infiltration in BM histology, %; median (range)	50 (50–75)	80 (40–90)	n.s.	50 (20–100)	n.s.	n.s.
Serum tryptase, µg/L; median (range)	209 (135–1300)	451 (113–1150)	n.s.	371 (41–1675)	n.s.	n.s.
Splenomegaly, *n* (%)	6 (100)	4 (57)	n.s.	25 (93)	n.s.	n.s.
Hepatomegaly, *n* (%)	2 (33)	2 (29)	n.s.	19 (70)	n.s.	n.s.
Lymphadenopathy, *n* (%)	3 (50)	3 (43)	n.s.	20 (74)	n.s.	n.s.
Additional SM and/or AHN relevant findings						
Leukocytes, ×10^9^/L; median (range)	9 (3.6–13.5)	6 (2–32)	n.s.	6.4 (1.3–66.1)	n.s.	n.s.
Monocytes, %; median (range)	4.8 (1.3–15)	5.4 (1–26)	n.s.	7.8 (1–42)	n.s.	n.s.
Eosinophils, %, median (range)	19.6 (1–33)	1 (0–28)	n.s.	2 (0–38)	0.0164	n.s.
Vitamin B12, ng/L; median (range)	300 (129–1526)	391 (303–6000)	n.s.	1597 (288–6000)	0.0379	n.s.
>180 ng/L, *n* (%)	5 (83)	7 (100)	n.s.	17 (100)	n.s.	n.s.
*S*/*A*/*R/D/N* mutation(s), *n* (%)	2 (33)	2 (29)	n.s.	19 (66)	0.0278	0.0135
Other additional mutations, *n* (%)	4 (67)	2 (29)	n.s.	15 (52)	n.s.	n.s.
Anaphylaxis, *n* (%)	1 (17)	1 (14)	n.s.	-	-	-
Treatment						
Midostaurin, *n* (%)	5 (83)	6 (86)	-	26 (90)	-	-
Cladribine, *n* (%)	3 (50)	6 (86)	-	13 (45)	-	-
Imatinib, *n* (%)	0 (0)	3 (43)	-	0 (0)	-	-
Follow-up, years, median (range)	1.8 (1–2)	0.9 (0.1–7.5)	n.s.	3.7 (0.04–11.8)	n.s.	n.s.
Death, *n* (%)	6 (100)	7 (100)	n.s.	18 (62)	n.s.	n.s.
Overall survival, median, years	1.7	0.9	n.s.	2.6	0.043	0.046

n.s., not significant; SM, systemic mastocytosis; AHN, associated hematological neoplasm; MCL, mast cell leukemia; MDS, myelodysplastic syndrome; MPN, myeloproliferative neoplasm; CLL, chronic lymphocytic leukemia; MM, multiple myeloma; chronic myelomonocytic leukemia; AML, acute myeloid leukemia; CEL, chronic eosinophilic leukemia; MC. mast cell; *S*/*A*/*R/D/N*, *SRSF2*, *ASXL1*, *RUNX1*, *DNMT3A*, *NRAS*.

**Table 3:** Morphological differences between *KIT*^neg.^ MCL ± AHN and SM-AHN patients.

#pat.	Age	Sex	Diagnosis	SM-Subtype	CD25	CD2	CD30	Differentiation	Tryptase [ng/mL]
8	54	M	SM-AHN	MCL	-	+	+	Well-differentiated	885
9	71	M	SM	MCL	-	+	+	Immature	1150
10	79	M	SM-AHN	MCL	-	+	+	Well-differentiated	180
11	63	M	SM	MCL	+	+	-	Immature	451
12	28	F	SM	MCL	+	n.d.	n.d.	Immature	202
13	27	M	SM	MCL	+	+	+	Immature	113
14	56	F	SM-AHN	MCL	n.k.	n.k.	n.k.	n.k.	631
15	64	F	SM-AHN	ISM	-	-	-	Well-differentiated	90
16	57	F	SM-AHN	ISM	+	-	-	Immature	96
17	59	M	SM-AHN	ISM	+	+	+	Immature	34
18	62	M	SM-AHN	ISM	+	-	-	Immature	31
19	82	M	SM-AHN	ISM	+	-	-	Immature	18

n.d., not done; n.k., not known; SM-AHN systemic mastocytosis with an associated hematological neoplasm; MCL, mast cell leukemia; M, male; F, female; +, positive; -, negative.

## Data Availability

The data presented in this study are available on request from the corresponding author (accurately indicate status).

## Appendix A

**Table A1:** Summary of relevant individual patient data.

#Pat.	Diagnosis	Age (Years)	Sex	Date of Dx	*KIT* Status	Additional Mutations	Tryptase (µg/L)	BM Infiltration (%)	A/T	M/E	AP (U/L)	Alb (g/L)	Death
#1	SM-B-NHL	66	M	2017	D816H	None	90	20	+/−	−/−	48	nk	Alive
#2	MCL	61	F	2011	D816Y	None	1300	50	−/−	−/+	162	36	Death
#3	MCL	58	M	2013	D816H	None	193	50	−/+	−/−	83	45	Death
#4	MCL-CEL	45	F	2015	D816H	*NRAS*	157	75	+/−	−/+	122	32	Death
#5	MCL-CMML	69	F	2011	D816Y	*TET2, CBL, SRSF2*	354	50	+/−	+/−	246	39	Death
#6	MCL-CEL	51	F	2015	D816H	*JAK2*	135	50	−/−	−/+	117	35	Death
#7	MCL	37	M	2018	D816Y	None	225	60	+/−	−/−	106	36	Death
#8	MCL-CLL	54	M	2018	Negative	None	885	90	−/−	−/−	81	41	Death
#9	MCL	71	M	2015	Negative	None	1150	50	+/+	−/−	95	36	Death
#10	MCL-MM	79	M	2013	Negative	*TET2*	180	50	−/+	−/−	59	39	Death
#11	MCL	63	M	2014	Negative	None	451	80	+/+	−/−	159	35	Death
#12	MCL	28	F	2018	Negative	None	202	40	+/+	−/−	131	20	Death
#13	MCL	27	M	2021	Negative	None	113	80	−/−	−/−	437	33	Death
#14	MCL-MDS/MPN	56	F	2021	Negative	*JAK2, DNMT3A, CSF3R*	631	90	+/+	−/+	210	31	Death
#15	SM-MDS	64	F	2017	Negative	*TET2*	90	10	−/+	−/−	69	41	Alive
#16	SM-AML	57	F	2013	Negative	*RUNX1*	96	20	−/−	−/−	62	39	Death
#17	SM-MF	59	M	2015	Negative	*JAK2, KRAS, TET2*	34	20	−/−	+/−	377	43	Alive
#18	SM-MDS	62	M	2022	Negative	*ASXL1, CSF3R, SRSF2*	31	35	−/−	−/−	107	38	Alive
#19	SM-MDS/MPN	82	M	2022	Negative	*FLT3-ITD, U2AF1, CBL, TET2*	18	10	−/−	+/−	45	36	Alive

Pat., patient number; M, male; F, female; Dx, diagnosis; BM, bone marrow; AP, alkaline phosphatase (normal value: 40-130U/L); Alb, albumin (normal value: 35–48 g/L); SM, systemic mastocytosis; MCL, mast cell leukemia; CMML, chronic myelomonocytic leukemia; CEL, chronic eosinophilic leukemia; B-NHL, B-cell non-Hodgkin lymphoma; MDS, myelodysplastic syndrome; MPN, myeloproliferative neoplasm; CLL, chronic lymphocytic leukemia; MM, multiple myeloma; AML, acute myeloid leukemia; MF, myelofibrosis; nk, not known; A/T: anemia Hb ≤ 10.0 g/dL (+), Hb > 10.0 g/dL (−), platelets < 100 × 10^9^/L (+), platelets ≥ 100 × 10^9^/L (−); M/E: monocytosis > 1 × 10^9^/L (+), ≤1 × 10^9^/L (−), eosinophilia > 1 × 10^9^/L (+), ≤1 × 10^9^/L (−), tryptase (normal value: <20 µg/L).

**Table A2:** Detailed localization of additional mutations on cDNA and protein level.

#Patient	Gene	Reference Sequence	cDNA Variant	Protein Variant
#4	*NRAS*	NM_002524.5	codon 61	codon 61
#5	*TET2*	NM_001127208.3	c.4210C>T	p.Arg1404*
	*CBL*	NM_005188.4	c.1169A>T	p.Asp390Val
	*SRSF2*	NM_001195427.2	c.284C>A	p.Pro95His
#6	*JAK2*	NM_004972.4	c.1849G>T	p.Val617Phe
#10	*TET2*	NM_001127208.3	c.2681_2687del	p.Ser894Tyrfs*25
#14	*JAK2*	NM_004972.4	c.1849G>T	p.Val617Phe
	*DNMT3A*	NM_022552.4	c.2645G>A	p.Arg882His
	*CSF3R*	NM_000760.4	not available	not available
#15	*TET2*	NM_001127208.3	c.1795C>T	p.Gln599*
	*TET2*	NM_001127208.3	c.2428C>T	p.Gln810*
#16	*RUNX1*	ENST00000344691	c.877C>T	p.Arg293*
#17	*JAK2*	NM_004972.4	c.1849G>T	p.Val617Phe
	*TET2*	NM_001127208.3	c.4546C>T	p.Arg1516*
	*TET2*	NM_001127208.3	c.4621C>T	p.Gln1541*
	*KRAS*	NM_004985.5	c.108A>G	p.Ile36Met
#18	*SRSF2*	NM_001195427.2	c.284C>T	p.Pro95Leu
	*ASXL1*	NM_015338.6	c.3514del	p.Ala1172Leufs*2
	*CSF3R*	NM_000760.4	c.2221C>T	p.Gln741*
#19	*TET2*	NM_001127208.3	c.1526C>G	p.Ser509*
	*TET2*	NM_001127208.3	c.5688G>T	p.Arg1896Ser
	*CBL*	NM_005188.4	c.1259G>T	p.Arg420Leu
	*U2AF1*	NM_006758.3	c.101C>T	p.Ser34Phe
